# Associations between the red blood cell distribution width and 30-day mortality in critically ill patients with delirium: a retrospective study using the MIMIC-IV database

**DOI:** 10.3389/fnagi.2025.1599858

**Published:** 2025-12-11

**Authors:** Songmei Ma, Lin Lin, Shuwen Zheng, Zhenjing Liu, Li Kong, Haiyun Wang

**Affiliations:** 1 Department of Anesthesiology, The First People's Hospital of Shangqiu, Shangqiu, Henan, China; 2 Department of Anesthesiology, The Third Central Clinical College of Tianjin Medical University, Tianjin, China; 3 Tianjin Key Laboratory of Extracorporeal Life Support for Critical Diseases, Tianjin, China; 4 Artificial Cell Engineering Technology Research Center, Tianjin, China; 5 Tianjin Institute of Hepatobiliary Disease, Tianjin, China

**Keywords:** delirium, red blood cell distribution width, prognosis, all-cause mortality, mimic iv

## Abstract

**Background:**

Red blood cell distribution width (RDW) is associated with increased mortality risk in critically ill patients. However, limited data are available on critically ill patients with delirium.

**Methods:**

Data from the Multiparameter Intelligent Monitoring in Intensive Care Database IV (MIMIC-IV) version 3.1 database were analyzed in this retrospective cohort research. The Confusion Assessment Method for the Intensive Care Unit (CAM-ICU) criteria were used to identify critically ill patients with delirium. The first RDW value was extracted within the first 24 h after intensive care unit admission. The endpoint was 30-day all-cause mortality. Multivariable Cox regression analysis was performed to examine the relationship between RDW and 30-day mortality. Age, sex, myocardial infarction, congestive heart failure, peripheral vascular disease, dementia, cerebrovascular disease, chronic pulmonary disease, diabetes, sepsis, and hemoglobin were considered for subgroup analysis.

**Results:**

A total of 10,600 patients were included, with a mean (standard deviation) age of 67.0 (16.7) years, of whom 6,007 (56.7%) were male patients. The increase in RDW was correlated with an increased risk of 30-day mortality in the Cox proportional regression analysis model (Hazard Ratio [HR] 1.04; 95% confidence interval [CI] 1.03–1.04). In comparison with the low-RDW group, the middle and high-RDW groups tended to have higher risks of 30-day all-cause mortality (HR, 1.54; [95% CI] [1.34–1.77]; HR 2.25 [95% CI] [1.96–2.58]; P trend < 0.0001). Restricted cubic spline (RCS) analysis demonstrated linear relationships between RDW and 30-day mortality. Subgroup analyses using the entire cohort also demonstrated higher 30-day all-cause mortality. Subgroup analyses across the entire cohort confirmed elevated 30-day all-cause mortality associated with higher red cell distribution width (RDW), with results aligning closely with the Cox proportional regression findings.

**Conclusion:**

An increase in RDW was associated with an increased risk of 30-day all-cause mortality in critically ill patients with delirium. RDW may serve as a valid indicator for assessing the severity and guiding the treatment of delirium patients in the ICU.

## 1 Introduction

Delirium is a common problem in intensive care unit (ICU) patients, characterized by an acute onset and fluctuating course of impaired cognitive functioning, so that a patient’s ability to receive, process, store, and recall information is strikingly impaired. [It represents the most frequent form of brain dysfunction in critically ill patients and is now recognized as a form of acute cerebral failure associated with severely poor outcomes]. It is associated with poor outcomes in ICU patients, including increased morbidity, prolonged length of stay, higher mortality rates, and Long-term Cognitive Decline (Ely et al., 2004; Thomason et al., 2005; Goldberg et al., 2020; Yan et al., 2023; Inouye et al., 1999). The Confusion Assessment Method for the Intensive Care Unit (CAM-ICU) is a widely validated and reliable tool for the diagnosis of delirium in the ICU setting (Ely et al., 2001), which was employed in this study to identify our patient cohort. Critically, emerging evidence indicates that even after hospital discharge, survivors of ICU delirium face a significantly elevated risk of long-term cognitive impairment and accelerated progression to dementia, highlighting the profound and lasting impact of this condition (Fong and Inouye, 2022; Pandharipande et al., 2013). The incidence of delirium in ICU settings ranges from 19 to 82% (Ely et al., 2004; Ely et al., 2001; Inouye et al., 2014; Su et al., 2016; Rosa et al., 2019), highlighting the urgent need for reliable biomarkers to enhance diagnostic precision and therapeutic strategies. Despite advances in critical care, delirium remains under-recognized and inadequately managed, underscoring the need for reliable biomarkers that can aid in risk stratification and prognosis.

Red cell distribution width (RDW), a routine hematological parameter reflecting red blood cell size variation, has emerged as a potential prognostic marker in various clinical contexts, including cardiovascular diseases, cancers, and critical illnesses (Kim et al., 2022; Seo et al., 2020; Kim et al., 2023). Elevated RDW indicates a severe imbalance in red blood cell regulation, involving both defective erythropoiesis and unusual red blood cell longevity, possibly linked to several metabolic disorders, such as shortened lifespan of telomere length, oxidative stress, inflammation, poor nutritional status, dyslipidemia, hypertension, erythrocyte fragmentation, and alteration of erythropoietin function, both of which are implicated in the pathophysiology of delirium.

Given the shared pathways of inflammation and physiological stress between elevated RDW and delirium and the established prognostic value of RDW in critical illness, we hypothesized that RDW would be associated with mortality in critically ill patients with delirium. However, data specifically examining this association in a delirium-focused cohort are limited.

This study aims to investigate the relationship between RDW levels and 30-day mortality in ICU patients diagnosed with delirium using data sourced from the MIMIC-IV database. By shedding light on this association, we strive to enhance the understanding of RDW’s prognostic value, potentially informing clinical decision-making and improving outcomes for critically ill patients. Through rigorous analysis, this study seeks to contribute meaningful insights that could guide therapeutic strategies and optimize care for patients experiencing delirium in the intensive care setting.

## 2 Materials and methods

### 2.1 Data sources and study population

The Multiparameter Intelligent Monitoring in Intensive Care Database IV (MIMIC-IV) version 3.1 provided data encompassing more than 90,000 ICU stays from 2008 to 2022 (Johnson et al., 2023). The database was operated by the Beth Israel Deaconess Medical Center. We completed the course Protecting Human Research Participants (Certification Number: 63322091), which is a National Institutes of Health web-based course. Our permission was approved by the Institutional Review Boards of the Massachusetts Institute of Technology (Cambridge, MA, United States) and the Beth Israel Deaconess Medical Center.

Patients were enrolled if they met the following criteria: (1) admitted to the hospital and the intensive care unit (ICU) for the first time; (2) RDW on the first day of admission to the ICU could be checked; (3) aged ≥ 18 years; (4) stayed in the ICU for more than 24 h; and (5) had a positive CAM-ICU screening result.

Patients were excluded from the analysis based on any of the following criteria.

**Figure 1:**
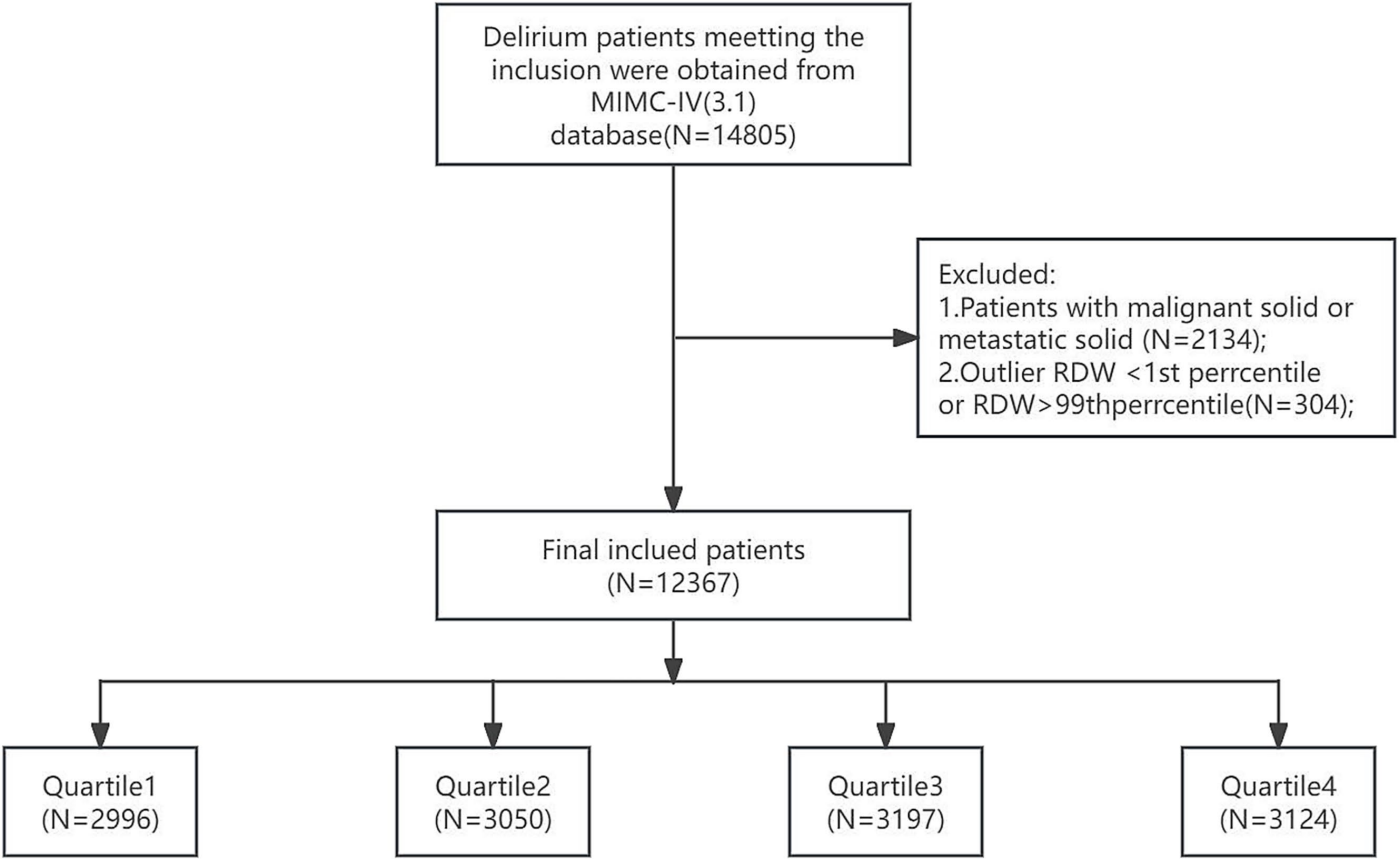
Flowchart of patient inclusion and exclusion criteria for the study. RDW, red cell distribution width. RDW quartiles: Quartile 1 (11.9–13.3), Quartile 2 (13.3–14.3), Quartile 3 (14.3–15.8), and Quartile 4 (15.9–23.3).

1. A documented diagnosis of malignant cancer.
2. Presence of metastatic solid tumors.
3. To mitigate the potential distortion from extreme values and enhance the robustness of the analysis, the RDW values were processed using a percentile-based Winsorization method. Specifically, values falling below the 1st percentile or above the 99th percentile were treated as missing data, thus retaining the central 98% of the distribution for the subsequent analysis. Finally, a total of 12,367 patients were enrolled in this study and grouped into four groups based on the quartiles of the RDW (Figure 1).

### 2.2 Data extraction

We employed Navicat Premium (Version 16.1.15) with a structured query language (SQL) to extract data documented on the first day following ICU admission. The dataset comprised patient demographics such as age, sex, and weight; clinical scores including the Charlson Comorbidity Index, the Simplified Acute Physiology Score (SAPS) II, and the Sequential Organ Failure Assessment (SOFA) score; laboratory tests such as hemoglobin, white blood cell (WBC) count, platelets, serum creatinine (Scr), and blood urea nitrogen (BUN); and information on comorbidities such as heart failure (HF), chronic obstructive pulmonary disease (COPD), chronic kidney disease (CKD), liver disease (mild or severe), dementia, diabetes, and cardiovascular disease. These details are presented in Table 1. We defined 30-day mortality as a measure of short-term prognosis and 365-day mortality as an indicator of long-term prognosis. Laboratory variables were collected only within the first 24 h of ICU admission.

**Table 1:** Baseline characteristics and clinical outcomes of the study population.

Variables	Total (*N* = 12,367)	RDWQ1 (*N* = 2,996)	RDWQ2 (*N* = 3,050)	RDWQ3 (*N* = 3,197)	RDWQ4 (*N* = 3,124)	*p*
Age, years	66.7 ± 17.2	62.3 ± 19.0	67.4 ± 17.0	69.2 ± 16.2	67.5 ± 16.0	< 0.001
Sex, *n* (%)						< 0.001
Female	5,459 (44.1)	1,179 (39.4)	1,299 (42.6)	1,504 (47)	1,477 (47.3)	
Male	6,908 (55.9)	1817 (60.6)	1751 (57.4)	1,693 (53)	1,647 (52.7)	
Weight, kg	82.2 ± 24.1	80.1 ± 20.7	81.8 ± 22.7	82.6 ± 25.2	84.2 ± 27.2	< 0.001
HR, bpm	85.8 ± 16.3	83.4 ± 15.3	85.3 ± 16.2	86.7 ± 16.5	87.7 ± 16.6	< 0.001
MBP mmHg	79.6 ± 11.0	81.8 ± 11.0	80.3 ± 10.8	79.2 ± 11.0	77.3 ± 10.7	< 0.001
CCI, scores	5.6 ± 2.8	4.5 ± 2.7	5.3 ± 2.6	5.9 ± 2.7	6.4 ± 2.7	< 0.001
SAPS II scores	39.8 ± 13.8	34.6 ± 12.6	38.6 ± 13.2	41.9 ± 13.6	43.7 ± 13.8	< 0.001
Sepsis, *n* (%)						< 0.001
No	4,041 (32.7)	1,335 (44.6)	1,057 (34.7)	915 (28.6)	734 (23.5)	
Yes	8,326 (67.3)	1,661 (55.4)	1993 (65.3)	2,282 (71.4)	2,390 (76.5)	
Hb, g/dL	10.2 ± 2.3	11.3 ± 2.1	10.7 ± 2.2	9.9 ± 2.2	8.9 ± 2.1	< 0.001
LOS ICU, days	7.0 ± 8.3	6.9 ± 8.3	7.0 ± 8.2	6.9 ± 7.7	7.3 ± 9.0	0.25
LOS hospital,days	16.3 ± 16.2	14.7 ± 15.6	15.3 ± 14.6	16.2 ± 14.9	18.8 ± 18.8	< 0.001
Vent-free 28 days	20.5 ± 11.1	23.2 ± 9.1	21.6 ± 10.3	20.2 ± 11.2	17.3 ± 12.8	< 0.001
Sedatives, *n* (%)						< 0.001
No	4,887 (39.5)	1,142 (38.1)	1,096 (35.9)	1,255 (39.3)	1,394 (44.6)	
Yes	7,480 (60.5)	1854 (61.9)	1954 (64.1)	1942 (60.7)	1730 (55.4)	
PLT/nL	166.0 (116.0, 226.0)	175.0 (136.0, 224.0)	170.0 (124.0, 223.0)	164.0 (110.0, 229.0)	152.0 (92.0, 229.0)	< 0.001
WBC/nL	13.5 (9.8, 18.3)	13.3 (10.0, 17.6)	13.6 (10.1, 17.9)	13.5 (9.7, 18.9)	13.5 (9.4, 18.9)	0.256
BUN, mg/dL	22.0 (15.0, 37.0)	17.0 (12.0, 24.0)	21.0 (14.0, 31.0)	25.0 (16.0, 41.0)	32.0 (19.0, 53.0)	< 0.001
Scr, mg/dL	1.1 (0.8, 1.8)	0.9 (0.7, 1.2)	1.1 (0.8, 1.5)	1.2 (0.9, 2.0)	1.5 (0.9, 2.6)	< 0.001
GLU, mg/dL	132.0 (111.0, 163.0)	129.1 (110.6, 156.4)	132.7 (112.4, 161.2)	134.3 (112.6, 167.5)	131.5 (108.0, 166.5)	< 0.001
Mor 30-day, (%)						< 0.001
No	9,933 (80.3)	2,693 (89.9)	2,578 (84.5)	2,534 (79.3)	2,128 (68.1)	
Yes	2,434 (19.7)	303 (10.1)	472 (15.5)	663 (20.7)	996 (31.9)	
Mor 90-day, (%)						< 0.001
No	9,171 (74.2)	2,584 (86.2)	2,420 (79.3)	2,332 (72.9)	1835 (58.7)	
Yes	3,196 (25.8)	412 (13.8)	630 (20.7)	865 (27.1)	1,289 (41.3)	
Mor 365-day, (%)						< 0.001
No	8,303 (67.1)	2,457 (82)	2,259 (74.1)	2057 (64.3)	1,530 (49)	
Yes	4,064 (32.9)	539 (18)	791 (25.9)	1,140 (35.7)	1,594 (51)	

Data are presented as mean ± SD or median (first quartile, third quartile) based on their distribution. RDW, red blood cell distribution width; CCI, Charlson comorbidity index; SAPS II, Simplified Acute Physiology Score II; HR, heart rate; bpm, beats/min; MBP, mean blood pressure; Hb, hemoglobin; PLT, platelet; WBC, white blood cell; BUN, blood urea nitrogen; Scr, creatinine; GLU, glucose; LOS, length of stay; ICU, intensive care unit; LOS, length of stay in hospital; Mor, Mortality. Significant differences were observed in most variables, as indicated by the *p-*value of <0.05.

### 2.3 Statistical analysis

The Kolmogorov–Smirnov test was employed to evaluate whether continuous variables followed a normal distribution. Continuous variables were presented as mean ± standard deviation if normally distributed or as median (interquartile range) if not. Categorical variables were expressed as frequencies (percentages). For the analysis of continuous variables, *t*-tests or ANOVA were used for normally distributed data, whereas the Kruskal–Wallis test or Mann–Whitney U test was applied for non-normally distributed data. Categorical variables were analyzed using the chi-square test or Fisher’s exact test, as appropriate. Kaplan–Meier survival analysis was conducted to assess the incidence of endpoint events across different RDW groups, with differences between groups evaluated using the log-rank test. We constructed five Cox proportional hazards models: Model 1 was unadjusted for any confounders; Model 2 was adjusted for age, sex, and weight; and Model 3 was further adjusted for CCI, SAPS II, and Sepsis-3. Model 4 was adjusted for age, sex, weight, CCI, SAPS II, Sepsis-3, hemoglobin, platelets, WBC, BUN, creatinine, and glucose. Model 5 was adjusted for age, sex, weight, CCI, SAPS II, Sepsis-3, hemoglobin, platelets, WBC, BUN, creatinine, glucose, sedatives, and ventilation. RDW was analyzed both as a continuous variable and as an ordinal categorical variable, with a P for the trend test performed to evaluate the linear association across categories. To examine whether a non-linear relationship existed between RDW and mortality, we employed a restricted cubic spline (RCS) regression model with four knots. If a non-linear association was detected, we further analyzed the data using a two-piecewise Cox model on either side of the inflection point. Furthermore, stratified analyses were conducted according to age (< 65 years or ≥ 65 years), sex (male, female), myocardial infarction, congestive heart failure, peripheral vascular disease, dementia, cerebrovascular disease, chronic pulmonary disease, diabetes, severe liver disease, sepsis, sedatives, and vent. For all analyses, a two-sided *p*-value less than 0.05 was considered to indicate statistical significance.

### 2.4 Baseline

A total of 12,367 patients who were admitted to the ICU and diagnosed with delirium according to the Confusion Assessment Method for the Intensive Care Unit (CAM-ICU) criteria (Ely et al., 2001), stratified into four quartiles (Q1–Q4) based on the variable of interest, were included in our study. The 30-day mortality for the included patients was 19.7%, and the 365-day mortality was 32.9%. Mortality rates at 30, 90, and 365 days increased significantly across quartiles (*p* < 0.001). With the TRDW increases, the age tended to be older, with the mean age increasing from 62.3 ± 19.0 years in Q1 to 67.5 ± 16.0 years in Q4; weight, HR, CCI, SAPS II, BUN, SCR, and glucose tended to be higher; and MBP, PLT, Hb, and Ventfree28 tended to be lower. In contrast, no significant trends were observed for ICU length of stay (ICU-LOS; *p* = 0.25).

### 2.5 Associations between the RDW and 30-day mortality

**Figure 2:**
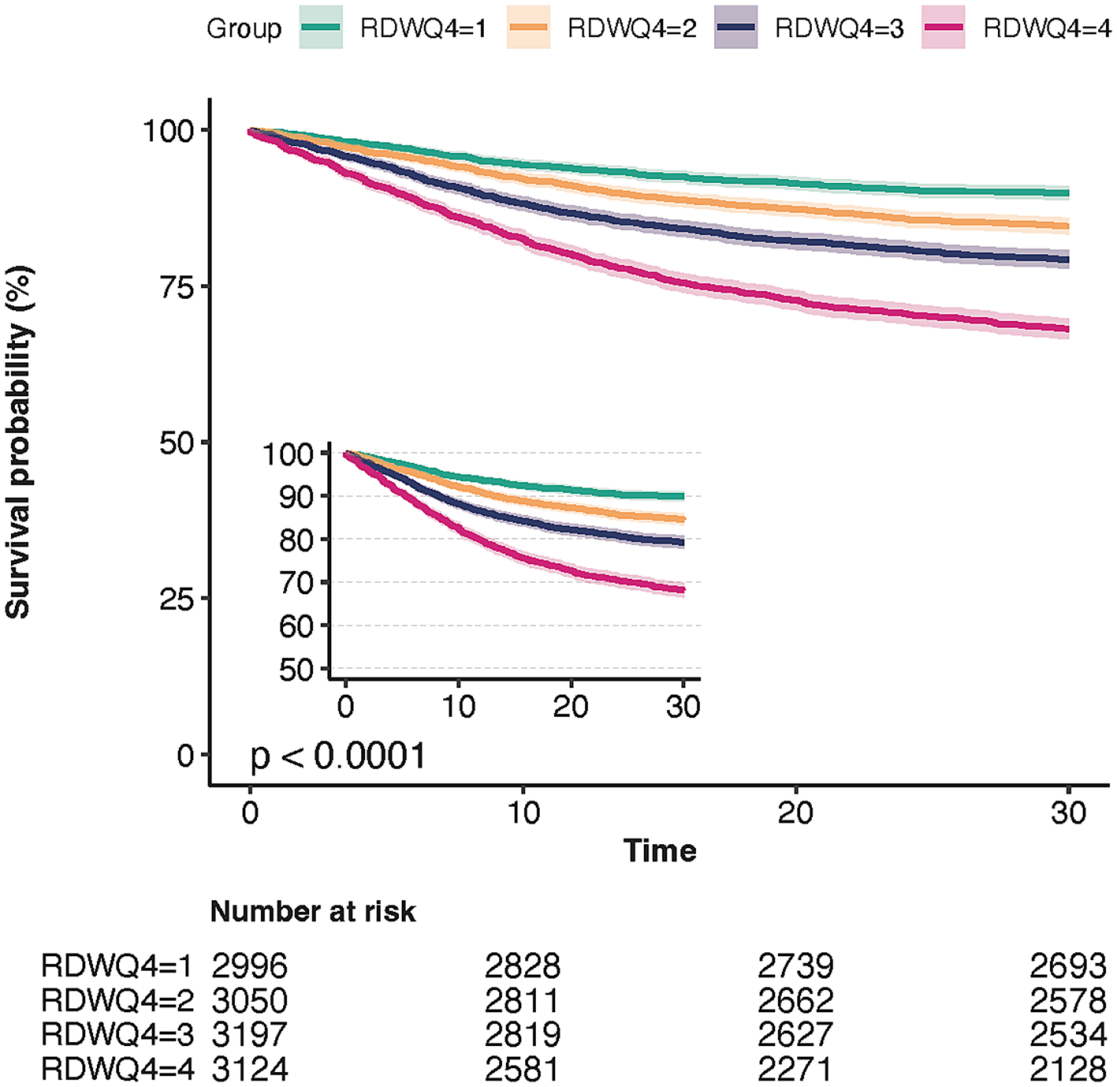
Kaplan–Meier curves of 30-day all-cause mortality stratified by RDW.

We used Kaplan–Meier survival analysis curves to examine the incidence of in-hospital and ICU mortality across the four RDW quartile groups (Figure 2), revealing that patients in the Q4 group had a significantly higher risk of 30-day mortality (log-rank *p* < 0.001).

To further investigate the relationship between RDW and mortality, we used Cox proportional hazards regression models (Table 2). The association between red blood cell distribution width (RDW) and 30-day mortality was evaluated using a multivariate Cox regression model. Five models were constructed with incremental adjustments for covariates to assess the independent effects of RDW on mortality. RDW was analyzed as a continuous variable or quartile, and a dose-dependent increase in mortality risk was observed.

**Table 2:** Multivariate cox regression to assess the association between RDW and 30-day mortality.

Variables	Model 1		Model 2		Model 3		Model 4	
	HR (95%CI)	*p*	HR (95%CI)	*p*	HR (95%CI)	*p*	HR (95%CI)	*p*
RDW	1.22 (1.2–1.24)	<0.001	1.16 (1.14–1.018)	<0.001	1.16(1.03–1.04)	<0.001	1.13 (1.11–1.15)	<0.001
RDWQ1	1(Ref)		1(Ref)		1(Ref)		1(Ref)	
RDWQ2	1.38 (1.19–1.59)	<0.001	1.2 (1.03–1.39)	0.016	1.18 (1.02–1.37)	0.025	1.17 (1.01–1.35)	0.037
RDWQ3	1.88 (1.64–2.16)	<0.001	1.43 (1.25–1.65)	<0.001	1.42 (1.23–1.63)	<0.001	1.37 (1.19–1.58)	<0.001
RDWQ4	3.26 (2.86–3.71)	<0.001	2.28 (2–2.61)	<0.001	2.24 (1.95–2.58)	<0.001	2.00 (1.74–2.3)	<0.001
Trend.test	1.5 (1.44–1.56)	<0.001	1.34 (1.28–1.39)	<0.001	1.33 (1.27–1.39)	<0.001	1.27 (1.22–1.33)	<0.001

HR, Hazard Ratio, CI, Confidence Interval. Model 1: covariates were adjusted for age, sex, and weight. Model 2: covariates were adjusted for age, sex, weight, CCI, SAPS II, and sepsis. Model 3: covariates were adjusted for age, sex, weight, CCI, SAPS II, sepsis, hemoglobin, platelets, WBC, BUN, creatinine, and glucose. Model 4: covariates were adjusted for age, sex, weight, CCI, SAPS II, sepsis, hemoglobin, platelets, WBC, BUN, creatinine, glucose, sedatives, and ventilation. RDW, red blood cell distribution width; HR, hazard ratio; CI, confidence interval.

Model 1 (adjusted for age, sex, and weight): The association between RDW and mortality remained significant, with HRs of 1.30 (Q2), 2.00 (Q3), and 3.51 (Q4; *p*-value for trend < 0.001).

Model 2 (further adjusted for CCI, SAPS II, and sepsis): The risk estimates were attenuated but remained significant, with HRs of 1.17 (Q2), 1.55 (Q3), and 2.30 (Q4; *p*-value for trend < 0.001).

Model 3 (hemoglobin, platelets, WBC, BUN, creatinine, and glucose): The fully adjusted model confirmed the independent association between RDW and mortality, with HRs of 1.18 (Q2), 1.54 (Q3), and 2.25 (Q4; *p*-value for trend < 0.001). Model 4 (fully adjusted for age, sex, weight, CCI, SAPS II, sepsis, hemoglobin, platelets, WBC, BUN, creatinine, glucose, sedatives and ventilation): The fully adjusted model confirmed the independent association between RDW and mortality, with HRs of 1.18 (Q2), 1.54 (Q3), and 2.25 (Q4; *p*-value for trend < 0.001).

### 2.6 The detection of non-linear relationships

The restricted cubic splines (RCS) curve analysis revealed a linear relationship between the RDW and 30-day mortality, between RDW standard deviation and the hazard ratio for 30-day mortality, and p for non-linearity = 0.96. The hazard ratio increased progressively with higher RDW values, with the reference point set at 14.3% (HR = 1.0). In the upper range of RDW = 23.3%, the hazard ratio reached approximately 5.0, indicating a five-fold increase in risk compared to the reference point. The 95% confidence interval remained narrow across the range of RDW values, underscoring the precision of this association. These findings suggest that RDW is a strong and independent predictor of adverse outcomes, with a dose-dependent relationship.

**Figure 3:**
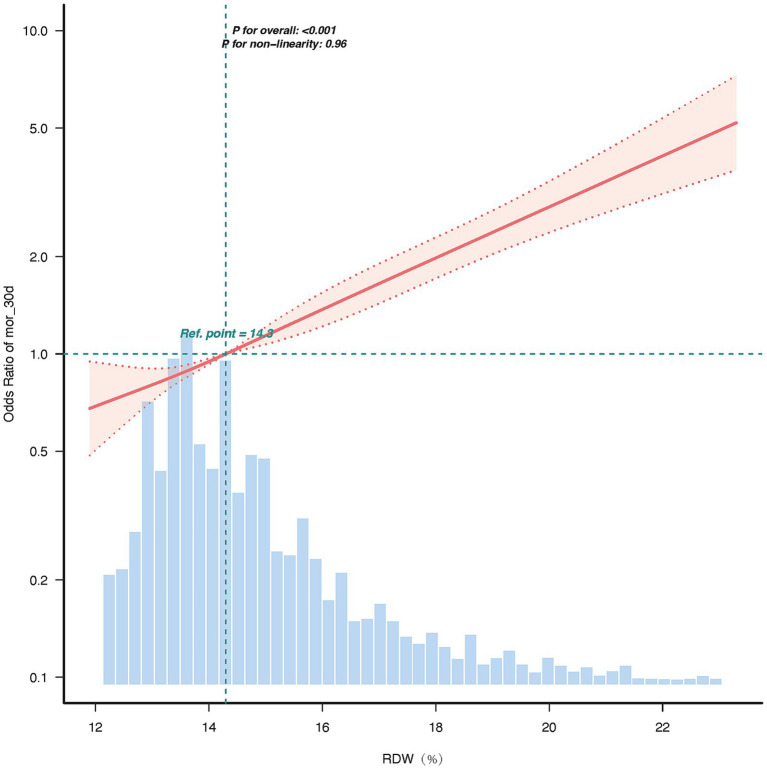
RCS of RDW with 30-day mortality.

As RDW increases, the hazard ratio rises significantly, indicating that higher RDW values are associated with a greater risk of adverse outcomes. The use of 4 knots placed at the default percentiles (5th, 35th, 65th, and 95th). The specific test statistic (χ^2^ = 0.09, df = 2) and *p*-value (*p* = 0.96) led us to accept the linearity assumption. The linearity of the relationship (p for non-linearity = 0.96) suggests that RDW is a robust and consistent predictor of risk across its range (Figure 3).

### 2.7 Subgroup analysis results

**Figure 4:**
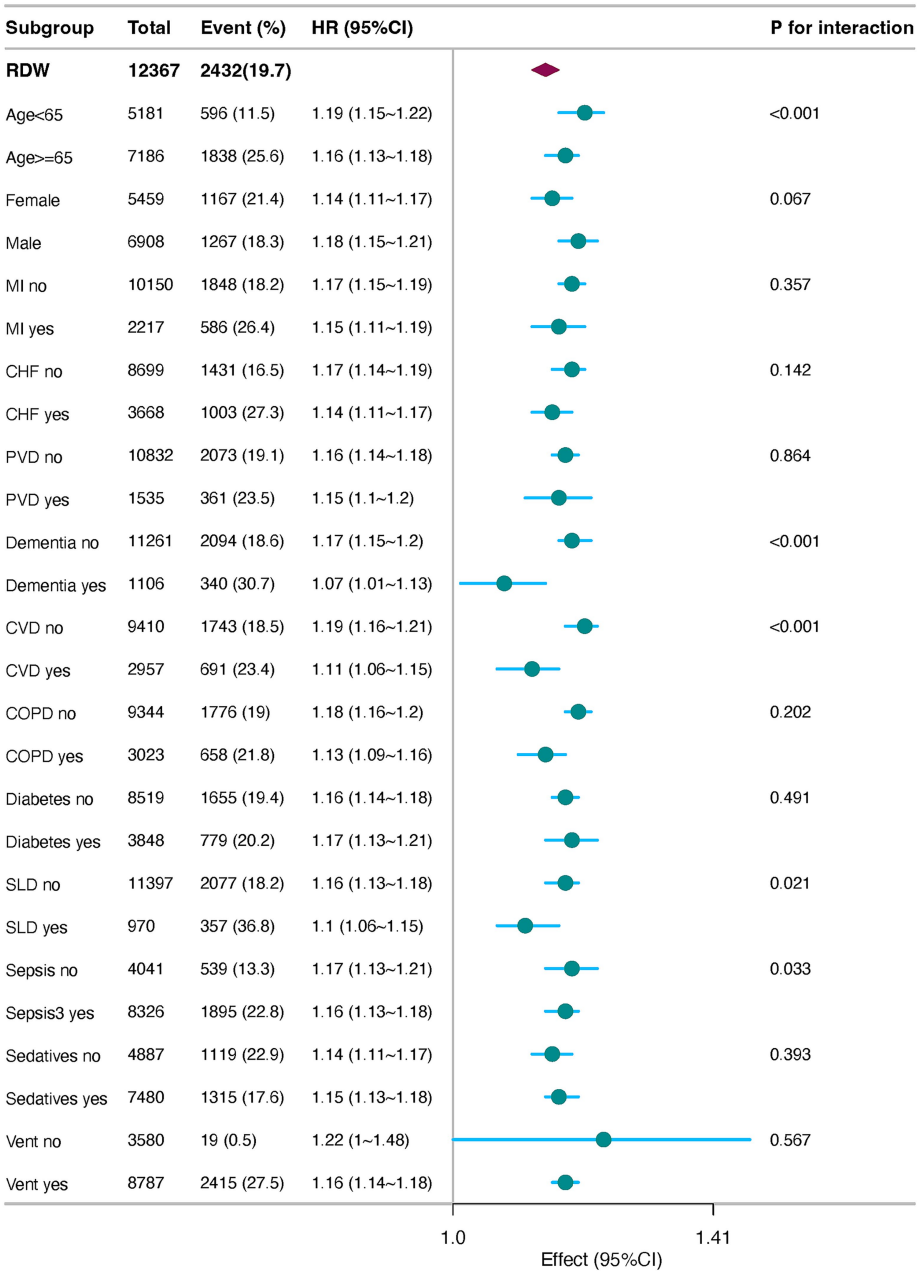
Forest plots of stratified analyses of RDW and 30-day all-cause mortality. MI, Myocardial Infarction; CHF, Congestive Heart Failure; PVD, Peripheral Vascular Disease; CVD, cerebrovascular disease; COPD, chronic pulmonary disease; SLD, severe liver disease; vent, ventilation.

In the subgroup analysis, the adjusted hazard ratios (adjusted HR) for RDW were consistently greater than 1 across all subgroups, with 95% confidence intervals excluding 1, indicating a significant positive association between RDW and event risk. Specifically, for patients aged <65 years, the adjusted HR was 1.33 (95% CI: 1.1–1.16), suggesting a 13% increase in event risk per standard deviation increase in RDW. Similarly, for patients aged ≥65 years, the adjusted HR was 1.11, indicating a comparable risk increase. In the sex subgroups, the adjusted HR was slightly higher in males (1.13, 95% CI: 1.11–1.16) than in females (1.11, 95% CI: 1.08–1.12), implying a potentially stronger effect of RDW on event risk in male patients. No significant differences were observed in the sepsis (Sepsis-3) and diabetes subgroups, with adjusted HR values ranging from 1.03 to 1.04, suggesting that these comorbidities did not significantly modify the association between RDW and event risk. The interaction *p*-values revealed significant effect modifications according to age (*p*-value for interaction = 0.011) and sex (*p*-value for interaction = 0.020). Specifically, the impact of RDW on event risk differed significantly between patients aged ≥65 years and <65 years, as well as between males and females. These findings underscore the importance of considering age and sex as potential modifiers of the association between RDW and clinical outcomes (Figure 4).

## 3 Discussion

This study aims to elucidate the association between RDW and mortality in critically ill patients with delirium and to evaluate the predictive value of RDW for prognosis. Our findings demonstrate that elevated RDW levels are a significant independent risk factor for 30-day mortality in these patients, even after adjusting for potential confounders. Our findings reveal a linear correlation between RDW elevation and mortality risk, providing novel insights into hematological biomarkers for neurological critical care outcomes.

RDW is a routine parameter in complete blood counts. Although RDW is affected to some degree by any type of anemia, researchers suggest that anemia is a risk factor for both dementia and cognitive impairment (Andro et al., 2013; Peters et al., 2008; Winchester et al., 2018; Qiang et al., 2023). C-reactive protein (CRP) and pro-inflammatory cytokines such as IL-6 have been consistently linked to the occurrence and duration of delirium in critically ill and postoperative patients (Leng et al., 2025; Mosharaf et al., 2025; Brummel et al., 2024). Sajjad et al. demonstrated that elevated levels of interleukin-8 (IL-8) in cerebrospinal fluid were significantly associated with delirium, highlighting the role of neuroinflammation (Spence et al., 2025; Denver et al., 2025). Similarly, researchers found that IL-1β levels were higher in delirium patients (Lopez-Rodriguez et al., 2021; Yu et al., 2023). The biological rationale is that systemic inflammation can disrupt the blood–brain barrier, activate microglia, and alter neurotransmission, leading to acute cognitive dysfunction (Brummel et al., 2024; Taylor et al., 2022; Stock et al., 2013; Hirsch et al., 2016). RDW fits directly into this paradigm, as pro-inflammatory cytokines (e.g., IL-6, TNF-*α*) are known to suppress erythropoiesis and alter red blood cell membrane integrity, directly leading to anisocytosis and elevated RDW (Zheng et al., 2019; Kim et al., 2018; Miao et al., 2019). DomingoAP (Pascual-Figal et al., 2009) persisted in that higher RDW levels were associated with worse long-term outcomes, regardless of hemoglobin levels and anemia status. RDW and inflammation-related indices based on complete blood counts (Lionte et al., 2021), such as hs-CRP, NLR, and MLR, were strongly associated with in-hospital mortality. IL-6 levels were significantly associated with an increase in RDW. Serum selenium may potentially mediate the effects on RDW through IL-6 (Lionte et al., 2021; Semba et al., 2010). A longitudinal study of 2,344 critically ill patients with COPD found that an increase in RDW was associated with an increased risk of 28-day all-cause mortality (Lan et al., 2024). A 9-year follow-up study involved 623 participants. Proteomics data showed that changes in IGFBP2 and C7 over 9 years mediated the association between changes in RDW and 6-year all-cause mortality. Cellular senescence may contribute to the association between RDW and mortality (Osawa et al., 2022). Several clinical studies have shown that an increase in RDW may be associated with other diseases, including acute pancreatitis, acute cerebral infarction, chronic kidney disease, gastrointestinal disorders, cancer, and cardiovascular disease (Osawa et al., 2022; Hammarsten et al., 2010; Kim et al., 2012; Chen et al., 2010; Ramírez-Moreno et al., 2013; Sánchez-Chaparro et al., 2010). At the same time, RDW in different diseases provides diagnostic and prognostic value (Parizadeh et al., 2019; Xanthopoulos et al., 2017; Sarkar et al., 2022; Felker et al., 2007).

Delirium is the most common manifestation of brain dysfunction in critically ill patients and is independently predictive of excess death, length of stay, cost of care, and acquired dementia (Stollings et al., 2021). The development of delirium is associated with a variety of factors, including infection, hypoxia, and metabolic disturbances (Inouye et al., 2014). Peripheral inflammation is a well-established trigger of delirium (Maclullich et al., 2008), and IL-1, IL-1β, IL-6, IL-8, tumor necrosis factor (TNF), and prostaglandin E2 (PGE2) stimuli trigger tissue macrophage and blood monocyte activation and secretion of inflammatory mediators (Sajjad et al., 2020; Cape et al., 2014; Hoogland et al., 2015). Although these molecules have a restricted ability to pass through the blood–brain barrier (BBB), they are also synthesized in the brain’s endothelial and epithelial cells and are directly secreted into the brain. They are primed if there is prior brain pathology, such as amyloid or earlier neurodegeneration; these microglia produce increased levels of these mediators, which affect both astrocytes and neurons (Podgoreanu et al., 2020). Cytokine-stimulated astrocytes produce increased levels of chemokines, contributing to the recruitment of monocytes and other immune cell populations to the brain. However, their activation also leads to the loss of metabolic support for neuronal energy metabolism. Thus, microglial-derived inflammatory mediators, such as IL-1β and TNF, directly affect neuronal function to produce both dysfunction and injury or cell death, which collectively may contribute to acute behavioral manifestations in delirium syndrome. Moreover, patients with delirium often experience restricted mobility, sleep deprivation (Stollings et al., 2021), and malnutrition (Volkert et al., 2024), all of which can further compromise immune function and overall physical condition, thereby increasing the risk of mortality. In our study, the 30-day mortality rate was significantly higher in the high RDW group of delirium patients compared to the low RDW group. This may be because elevated RDW levels reflect more severe states of inflammation, anemia, hypoxia, and cellular senescence, which interact with the pathogenesis of delirium to worsen the clinical course and outcomes of patients. Therefore, elevated RDW can be construed as a hematological manifestation of the same systemic inflammatory state that drives neuroinflammation and delirium.

Elevated RDW is a recognized marker of sustained systemic inflammation, characterized by elevated levels of pro-inflammatory cytokines (e.g., IL-6, TNF-*α*). In critically ill patients, these cytokines can compromise the integrity of the blood–brain barrier (BBB), facilitating their translocation into the central nervous system (Liu and Zhang, 2025). Once within the brain parenchyma, they activate microglia, the brain’s resident immune cells, triggering neuroinflammation. This state impairs synaptic function, reduces cerebral plasticity, and exacerbates neuronal injury, providing a plausible pathway linking high RDW to the severity and poor outcomes of delirium (Hickman et al., 2018). Furthermore, this pervasive systemic inflammatory state can directly worsen the severity of concurrent organ failures (e.g., septic shock and acute respiratory distress syndrome), contributing to multi-organ dysfunction and death. Linking RDW, Anemia, and Cerebral Oxygen Delivery: While RDW is distinct from hemoglobin levels, a high RDW often coincides with anemia of chronic disease. The conjunction of anemia, reduced oxygen-carrying capacity, and high RDW reflecting underlying inflammation and oxidative stress may create a double-hit scenario (Weiss and Goodnough, 2005). This could lead to suboptimal cerebral oxygen delivery in a brain already vulnerable to critical illness and neuroinflammation, potentially precipitating ischemic neuronal damage and impeding recovery, thereby increasing mortality risk. Future studies measuring cerebral oxygenation or biomarkers of neuronal injury, neurofilament light chain, alongside RDW, could help to validate this hypothesis.

RDW, as a marker of a pre-existing pro-inflammatory or chronic inflammatory state, can be used as a predictor of delirium progression. In general, a higher RDW is associated with increases in the erythrocyte sedimentation rate (ESR) and the inflammatory markers IL-6, C-reactive protein, and receptors for TNF I and II (Lippi et al., 2009; Lee and Kim, 2010). Pro-inflammatory cytokines found in patients with SIRS, including TNF-*α*, IL-6, and IL-1β, suppress erythrocyte maturation, allowing newer, larger reticulocytes to enter the peripheral circulation and increase RDW. Furthermore, pro-inflammatory cytokines can have direct inhibitory effects on the half-life of red blood cell circulation and deformability of the red blood cell membrane, which in turn can manifest as an increase in RDW. These observations provide support for the biological plausibility of RDW as a marker of inflammation in critical illness. The association between elevated RDW and poor outcomes is not unique to delirium but has been consistently reported in other neurological conditions, such as ischemic stroke (Feng et al., 2017) and Alzheimer’s disease (Huang et al., 2022). This consistency across diverse brain pathologies strengthens the argument that RDW is not a disease-specific marker but rather a proxy for a maladaptive systemic physiological state (e.g., chronic inflammation, oxidative stress, and nutritional deficiency) that negatively impacts brain resilience and recovery capacity. The acutely vulnerable brain during delirium may be particularly susceptible to the deleterious effects of this state. Subgroup analysis revealed that elevated red cell distribution width (RDW) was consistently and significantly associated with increased 30-day mortality across all patient subgroups (adjusted HR > 1), underscoring its robustness as a prognostic marker for delirium. Notably, the effect was significantly stronger in patients aged <65 years (HR 1.33, 95% CI 1.26–1.41) compared to those aged ≥65 years (HR 1.11, 95% CI 1.09–1.13; P for interaction <0.001), suggesting that RDW elevation may represent a more acute and severe pathological insult in younger, typically resilient individuals. The universal association across comorbidities implies a shared underlying pathophysiology, likely centered on systemic inflammation and oxidative stress, which concurrently drive RDW elevation, neuronal injury in delirium, and multi-organ failure. This finding suggests that RDW is an integrative hematological marker of global physiological derangement. Future research should prioritize investigating the mechanisms behind heightened vulnerability in younger patients and exploring the utility of RDW for risk stratification in this specific demographic. Furthermore, increased RDW mirrors a profound deregulation of erythrocyte homeostasis involving both impaired erythropoiesis and abnormal red blood cell survival, which may be attributed to a variety of underlying metabolic abnormalities, such as shortening of telomere length, oxidative stress, inflammation, poor nutritional status, dyslipidemia (Lippi et al., 2013), hypertension, erythrocyte fragmentation, and alteration of erythropoietin function.

Despite the robust associations observed, this study has limitations that warrant consideration. First, the retrospective design precludes a definitive causal inference between RDW and delirium-related mortality. Although we adjusted for extensive confounders, unmeasured variables or residual confounding factors may persist. Second, RDW was measured only once within 24 h of ICU admission, limiting insights into dynamic changes and their temporal relationship with delirium progression. Third, the lack of delirium subtype data and neurobiological markers hinders mechanistic exploration. Future prospective studies should integrate serial RDW measurements, delirium phenotyping, and multi-omics approaches to disentangle causality. Nonetheless, this study underscores the clinical utility of RDW in risk stratification, urging further investigation of its role within the delirium pathophysiology spectrum.

## 4 Conclusion

This cohort study suggests that an increase in RDW is associated with a higher risk of 30-day all-cause mortality in critically ill patients with delirium.

## Data Availability

The original contributions presented in the study are included in the article/Supplementary material, further inquiries can be directed to the corresponding author.
