# Supplementary material for: Associations between the red blood cell distribution width and 30-day mortality in critically ill patients with delirium: a retrospective study using the MIMIC-IV database
Source: Front Aging Neurosci. 2025 Dec 11;17:1599858. doi: 10.3389/fnagi.2025.1599858 (PMC12738919; doi:10.3389/fnagi.2025.1599858)
Supplement: Supplementary file 1 [file Table_1.docx]

**​STROBE Statement—Checklist of Items for Cohort Studies​**

| Section & Item | Item Description | Location in Manuscript (or Response) |
| --- | --- | --- |
| ​Title and Abstract​ |  |  |
| 1 | ​​(a) Indicate the study's design with a commonly used term in the title or the abstract​ | ​Title:​​ "...a retrospective study..." |
|  | ​​(b) Provide in the abstract an informative and balanced summary of what was done and what was found​ | ​Abstract:​​ The abstract clearly summarizes the background, methods, results, and conclusions. |
| ​Introduction​ |  |  |
| 2 | ​Background/rationale​ | ​Introduction, Paragraphs 1-4:​​ Provides comprehensive background on delirium and RDW, establishing the rationale for the study. |
| 3 | ​Objectives​ | ​Introduction, Final Paragraph:​​ "This study aims to investigate the relationship between RDW levels and 30-day mortality in ICU patients diagnosed with delirium..." |
| ​Methods​ |  |  |
| 4 | ​Study design​ | ​Abstract & Methods 2.1:​​ Clearly stated as a retrospective cohort study. |
| 5 | ​Setting​ | ​Methods 2.1:​​ The MIMIC-IV database (v3.1), Beth Israel Deaconess Medical Center, ICU admissions from 2008-2022. |
| 6 | ​Participants​ | ​Methods 2.1:​​ Inclusion and exclusion criteria are explicitly listed. A flow diagram (Figure 1) is also provided. |
| 7 | ​Variables​ | ​Methods 2.2 & Statistical Analysis:​​ Outcomes (30-day mortality), exposure (RDW), predictors, and confounders (age, sex, comorbidities, lab values, scores) are all clearly defined. |
| 8 | ​Data sources/ measurement​ | ​Methods 2.1 & 2.2:​​ Data source is MIMIC-IV. Tools used: CAM-ICU for delirium, Navicat Premium with SQL for extraction. Laboratory variables were collected within the first 24 hours. |
| 9 | ​Bias​ | ​Methods 2.1:​​ Addressed by using Winsorization to handle extreme RDW values (trimming 1st and 99th percentiles). Multiple statistical adjustments were made for confounding. |
| 10 | ​Study size​ | ​Methods 2.1 & Results:​​ The initial and final sample sizes after applying inclusion/exclusion criteria are provided (n=12,367 included). |
| 11 | ​Quantitative variables​ | ​Methods 2.1 & Statistical Analysis:​​ RDW was analyzed as both a continuous variable and a categorical variable (quartiles). The rationale for quartiles is implied by the study design. |
| 12 | ​Statistical methods​ | ​Statistical Analysis Section:​​  ​​(a)​​ Describes all methods: t-tests, ANOVA, chi-square, Kaplan-Meier, Cox regression models, RCS.  ​​(b)​​ Describes subgroup analysis.  ​​(c)​​ Missing data for Winsorized RDW values were treated as missing, but handling of missing data for other variables is not explicitly stated.  ​​(d)​​ Loss to follow-up is not applicable for mortality endpoints in this database context.  ​​(e)​​ Sensitivity analysis is not explicitly mentioned, but the use of multiple adjusted models and RCS serves a similar purpose. |
| ​Results​ |  |  |
| 13 | ​Participants​ | ​Methods 2.1 & Results (Baseline):​​  ​​(a)​​ Numbers at each stage are shown in the flow diagram (Figure 1).  ​​(b)​​ Reasons for exclusion are given (cancer, metastatic tumors, extreme RDW values).  ​​(c)​​ A flow diagram is provided (Figure 1). |
| 14 | ​Descriptive data​ | ​Table 1:​​  ​​(a)​​ Comprehensive table of characteristics by RDW quartiles is provided.  ​​(b)​​ The number of participants with missing data is not explicitly stated for each variable.  ​​(c)​​ Follow-up time is defined by the fixed outcomes (30-day, 365-day mortality). |
| 15 | ​Outcome data​ | ​Table 1 & Results:​​ Reports numbers of outcome events (30d, 90d, 365d mortality) for each RDW group. |
| 16 | ​Main results​ | ​Table 2 & Text:​​  ​​(a)​​ Provides both unadjusted (Model 1) and adjusted estimates (Models 2-4) with 95% CIs. Confounders adjusted for are clearly listed for each model.  ​​(b)​​ Category boundaries for RDW quartiles are provided (Q1: 11.9-13.3, etc.).  ​​(c)​​ Absolute risks are presented in Table 1 (mortality percentages). |
| 17 | ​Other analyses​ | ​Subgroup Analysis & RCS Sections:​​ Reports results of subgroup analyses (Figure 4) and the restricted cubic spline analysis (Figure 3) for non-linearity. |
| ​Discussion​ |  |  |
| 18 | ​Key results​ | ​Discussion, Paragraph 1:​​ Summarizes the key finding that elevated RDW is an independent risk factor for mortality. |
| 19 | ​Limitations​ | ​Discussion, Paragraph 5:​​ Discusses limitations: retrospective design (causality), single RDW measurement, lack of delirium subtype data. |
| 20 | ​Interpretation​ | ​Entire Discussion:​​ Provides a cautious interpretation of results, discusses potential mechanisms, and places findings in the context of existing literature. |
| 21 | ​Generalisability​ | The setting (single, large US hospital database) is described, but a specific discussion on generalizability to other populations is limited. |
| ​Other Information​ |  |  |
| 22 | ​Funding​ | ​Funding Statement:​​ "No funding was provided for this study." |

​**Note:​**​ The items marked with an asterisk (*) typically request information to be given separately for exposed and unexposed groups. In this study, the "exposure" (RDW) is a continuous/ordinal variable, so the results are presented by exposure quartiles (Q1-Q4), which fulfills the spirit of this guideline.
